# Assessing the daily association of sleep hygiene behaviours with sleep: A between and within persons approach

**DOI:** 10.1007/s10865-023-00448-0

**Published:** 2023-09-13

**Authors:** Thomas McAlpine, Barbara Mullan, Patrick J. F. Clarke

**Affiliations:** 1 enAble Institute, Curtin University, Bentley, WA 6102 Australia; 2 School of Population Health, Curtin University, Bentley, WA 6102 Australia

**Keywords:** Sleep, Sleep hygiene, Linear mixed modelling, Linear mixed effects

## Abstract

Sleep hygiene behaviours are recommendations given to both clinical and non-clinical populations with a focus on modifying behaviours to maximise sleep outcomes. However, methodological issues present in sleep hygiene research make it difficult to conclusively determine the impact of each behaviour. This study aimed to address these issues by adopting a two-week, repeated measures design which incorporated objective sleep measures and used linear mixed effect modelling to assess the daily association of a wide range of sleep hygiene behaviours on sleep in a non-clinical, university sample. Between-persons effects revealed that bedtime and frequency of daytime napping, alcohol use, and social media use were negatively related to sleep duration while waketime and frequency of too much water consumption were positively related to sleep duration. Within-person effects revealed that later than usual bedtime, earlier than usual waketime, no sunlight exposure, poor ventilation, having an unpleasant conversation before bed were negatively associated with sleep duration whereas using alcohol to deliberately help full asleep was positively related to sleep duration. In contrast, disproportionately more behaviours were not significantly related to either sleep outcome, only some of which could be explained by individual differences, which suggests that more research is needed to determine the conditions under which these behaviours affect sleep, if at all.

## Introduction

Insufficient sleep is a pervasive issue that impacts substantial proportions of the general population, with estimates that typically range from 20 to 35% of the population at any given time (Grandner, 2019; Hillman & Lack, 2013; Liu et al., 2013). Those who experience insufficient sleep include people with sleep disorders, but also those without clinical diagnoses who experience issues like difficulty falling asleep, getting fewer than seven hours of sleep a night, and waking frequently during the night (Hillman & Lack, 2013). Whether at or below clinical levels, insufficient sleep can contribute to a range of issues with physical and psychological functioning, which place a large burden on individuals, societies, and indeed economies around the world (e.g., Hillman et al., 2018).

To combat insufficient sleep in the general population, many sleep hygiene practices have been recommended to increase sleep quality (Brown et al., 2002; Lacks & Rotert, 1986; Mastin et al., 2006). Sleep hygiene practices generally consist of modifiable behaviours which when performed or avoided are believed to positively impact sleep and include activities like going to bed at the same time each night, avoiding exercise too close to bed, and getting sufficient exposure to sunlight/outdoor light during the day (Brown et al., 2002; Mastin et al., 2006; Yang et al., 2010). Despite being one of the most commonly recommended practices for improving sleep across various fields for the last two decades (Bloom et al., 2009; Stepanski & Wyatt, 2003; Sun et al., 2019), sleep hygiene components of CBT-I interventions for those with insomnia are generally the least efficacious (Taylor & Pruiksma, 2014; Taylor et al., 2010), and although some interventions which employ sleep hygiene behaviour change techniques have shown some success in improving sleep outcomes in general populations (Groenewold et al., 2019; Mairs & Mullan, 2015), others have shown efficacy in only certain types of sleep outcomes (e.g., sleep duration but not sleep efficiency; Anderson et al., 2022). This may be in part due to the myriad of issues that present in the measurement of sleep and determination of what constitutes a sleep hygiene behaviour.

An examination of three commonly used sleep hygiene scales shows great variation in the number of behaviours assessed (e.g., 13–30; Lacks and Rotert, 1986; Mastin et al., 2006; Yang et al., 2010). This speaks to the first key issue within sleep hygiene research; the lack of consensus as to which behaviours are important in affecting sleep, and therefore which behaviours ought to be included in sleep hygiene scales. Another measurement issue is the use of different parameters to define the performance of some behaviours. For example, recommendations generally align with the abstinence of alcohol before bed, but recommendations about the quantity, or the timing (i.e., how many hours before bed) differ markedly between scales (Brown et al., 2002; Mastin et al., 2006; Yang et al., 2010). Finally, the omission of other controllable behaviours, like exposure to blue light, which emerging evidence suggests can impact sleep (Burkhart & Phelps, 2009), results in an incomplete set of recommendations by which to improve sleep.

In addition to the aforementioned issues, research assessing the impact of individual behaviours within scales have often reported conflicting findings. For example, behaviours such as daytime napping have demonstrated negative (Mograss et al., 2022; Werth et al., 1996), and no impacts on sleep (Pilcher et al., 2001). Similarly, long-standing recommendations advise against exercising ‘before bedtime’, however much of the literature suggests that this impact is positive unless the exercise is vigorous and specifically within one hour of bed (Stutz et al., 2019). Overall, extensive inconsistencies across sleep hygiene behaviours suggests that the value of certain sleep hygiene recommendations may be questionable without further consideration of potential factors that facilitate their impact on sleep.

One such contributing factor relates to the measurement of sleep outcomes. Many studies that assess sleep hygiene rely solely upon subjective measures of sleep quality (e.g., Brick et al., 2010; Knufinke et al., 2018; Peach et al., 2016), despite the evidence suggesting that self-report estimates often differ from objective measures such as actigraphy (Aili et al., 2017; Jackowska et al., 2011), and polysomnography (Cudney et al., 2022). Therefore, the use of subjective measures alone may explain some of the discrepant associations between sleep hygiene behaviours and sleep that are prevalent in the extant literature, and a combination of both objective and subjective measures may be most beneficial to detect true effects.

A second methodological issue pertains to the cross-sectional nature of assessed sleep hygiene behaviour impact in research. Typically, research will assess general performance of behaviours over retrospective periods of time and attempt to find associations with overall sleep quality either concurrently or at a single time point in the future (e.g., Mastin et al., 2006; Peach et al., 2016; Yang et al., 2010). While this research is critical for identifying preliminary associations, a more direct understanding of their effect can be garnered by assessing the immediate impact of given behaviours on sleep recorded on a daily basis. Specifically, this permits examination of the discrete impact of engaging in any particular sleep hygiene behaviour on sleep for any given night. To the best of our knowledge, no research has assessed the daily impact of multiple sleep hygiene behaviours over time.

A final methodological issue concerns the potential influence of sleep hygiene behaviours within and between specific populations examined. Specifically, the effects of certain sleep hygiene behaviours may depend on individual differences, which could obscure relationships obtained at the overall population level. One potential example is the tolerance levels from sustained use of caffeine, which may render its impact on sleep negligible for regular users, but not for occasional users (Irish et al., 2015). In a similar way, the effect of other sleep hygiene behaviours may also vary based on individual differences in tolerance, habituation, or sensitisation to the specific sleep hygiene behaviour in question. Therefore, being able to account for these individual differences may allow for more complete understanding of the effect of sleep hygiene on sleep.

Overall, both methodological and individual differences may help provide an explanation for the inconsistent findings when assessing sleep hygiene impact. As such, a more rigorous examination of the effect of these sleep hygiene behaviours is warranted, so that sound advice can be given to general populations to improve sleep and reduce associated adverse outcomes of sleep insufficiency. Therefore, the aim of the present research was to assess the impact of a highly inclusive set of 32 sleep hygiene behaviours on sleep, using both objective and subjective measures, over a fourteen-day period. In addition, the secondary aim was to assess the inter-individual variation of these impacts to ultimately gain a more complete understanding as to the effect of each behaviour on sleep. Given the broad range of current sleep hygiene behaviours, specific hypotheses based on current evidence for each behaviour are not posited. Rather, the research will permit the determination of the degree of association between each individual behaviour and indicators of sleep.

## Methods

### Procedure and participants

Seventy-four participants were recruited from an Australian undergraduate university student pool over a four-month period between August and October 2021 (with no COVID-19 restrictions in place). University populations typically experience greater sleep disturbances (Azad et al., 2015; Gaultney, 2010; Orzech et al., 2011) and high variation in both their sleep and daily social schedules (Cai et al., 2017; Carney et al., 2006), which made them a suitable target for assessing the wide range of sleep hygiene behaviours on sleep. Participants completed baseline surveys upon induction and were given an accelerometer to measure their sleep activity over the next 14 nights resulting in 1036 individual daily observations. At baseline, participants provided demographic information and indicated which sleep hygiene behaviours they never performed to minimise completion of redundant items. It was reinforced that they should select ‘Yes’ for behaviours that they do sometimes, even if it was rare.

For the remainder of the study, a personalised daily questionnaire was sent via SMS to each participant each morning at a time that corresponded to their identified usual wake-up time. In it they were asked questions about their previous night’s sleep and were also asked about which sleep hygiene behaviours they had engaged in on the previous day, which included a section to add any additional comments about their sleep. Overall, participants completed 95.6% of these daily surveys and the fewest number completed was 10. Missingness for each variable ranged from 0 to 12.7% with the overall extent of missingness equalling 4.5%. Participants were awarded course credit for participating.

### Measures

#### Sleep

Objective sleep outcomes were assessed using ActiGraph GT9X Link© (ActiGraph LLC., Pensacola, FL, USA) monitors, worn on the non-dominant wrist over the 14-day duration of the study. Monitors were set to record data at sample rate of 30 Hz and Epoch value set at 60 s, using three axes to collect data. Using ActiLife (v6.13.4) software, the Tudor-Locke algorithm was used to detect sleep periods, with a minimum non-zero epoch set to 15. The Cole-Kripke algorithm was used to analyse these sleep periods for each participant. Sleep outcomes collected from actigraphy included sleep efficiency (the percentage of time spent asleep compared to the total time in bed), sleep-onset latency (the time taken to fall asleep after the first attempt), number of awakenings (the number of awakenings recorded between the start of sleep and the final awakening), mean length of awakening time (the average amount of time these awakenings lasted), and wake after sleep onset (the amount of time, in minutes, spent awake between the start of sleep and the final awakening), although sleep duration (the total time spent asleep between the start of sleep and the final awakening) was chosen as the sleep outcome of interest due to its more established association with all-cause mortality (Cappuccio et al., 2010).

A consensus sleep diary (Carney et al., 2012) was included in each daily questionnaire which contained questions that aligned with the objective sleep measures (e.g., “What time did you try to go to sleep?”, “In total, how long did you sleep for?”). This was done to help validate data from actigraphy. For instance, similar to previous research (Quante et al., 2018), sleep periods obtained from actigraphy were inspected and adjusted to ensure they aligned with both self-reported sleep information and sharp spikes/drops in activity. Likewise, if the algorithm detected more than one sleep period during the time specified by a participant’s sleep diary, periods were combined into one (Lee et al., 2018).

#### Sleep hygiene

Sleep hygiene behaviours present in currently used scales (Lacks & Rotert, 1986; Mastin et al., 2006; Yang et al., 2010) were considered for inclusion in the list. A degree of overlap between items allowed for some to be collapsed which reduced the number of sleep hygiene behaviours to be examined and subsequently decreased participant burden. Prior research (McAlpine et al., in press) was used to identify candidate items for exclusion and a final set of 32 behaviours was established for the present study. For a full list of sleep hygiene behaviours asked at baseline please refer to Table 1.

**Table 1:** List of sleep hygiene behaviours assessed

Sleep Hygiene Behaviour
Worrying about falling asleep while in bed
Having an unpleasant conversation before bed
Falling asleep with music or the TV on
Pondering unresolved matters before bed
Checking the time in the middle of the night
Worrying about sleep during the daytime
Engaging in vigorous intensity exercise (e.g., boxing, running > 5 mph / > 8 kph, swimming, soccer, jumping rope, etc.)
Napping during the day
Having any exposure to sunlight or outdoor light during the day
Engaging in moderate intensity exercise (e.g., briskly walking, lightly cycling, cleaning heavily, etc.)
Going to bed hungry
Going to bed thirsty
Having caffeine before bed
Consuming any other stimulating substances before bed (e.g., nicotine)
Having alcohol before bed
Drinking too much water before bed
Eating too much food before bed
Going to sleep in an environment that is too noisy (or quiet)
Going to sleep in an environment that is too bright (or dark)
Going to sleep in an environment that is too humid (or dry)
Going to sleep in an environment that is poorly ventilated
Going to sleep on an uncomfortable bed or pillow/s?
Having sleep interrupted by a partner (e.g., snoring, changing positions)
Use a screen (phone, computer, laptop, TV, etc.) before bed
(*IF YES*) - Using social media on this device (e.g., Facebook, Instagram, Snapchat, Twitter, Tik Tok)
Engaging in activities of high concentration before bed
Feeling stressed out or in another emotional state before bed (e.g., angry, upset)
Using sleep medications to help fall asleep
Drinking alcohol with the intention of using it to help fall asleep
Having sleep interrupted by pets
Going to bed at a different time than usual
Waking up at a different time than usual

Sleep hygiene was assessed each morning by asking participants which behaviours they had engaged in on the previous day. Participants could indicate “Yes”, or “No” for each item, and for behaviours that required more detail, follow-up prompts were asked if “Yes” was selected. For example, if participants indicated that they had exercised vigorously on the previous day, they were also asked for how long, and how close to bedtime the exercise was. Within-persons components were extracted from actigraphy determined bed and waketimes and used for the routine-related sleep hygiene behaviours (inconsistent bedtime/waketime).

#### Demographics

Age, gender, and prior diagnosis of sleep disorder were assessed by self-report.

#### Statistical analyses

Missing data was handled using multiple imputation, a method suitable for linear mixed modelling (Huque et al., 2018), provided any clustering of data is accounted for. The jomo package (Quartagno & Carpenter, 2022) was used to impute missing data for both continuous and categorical variables to create 10 imputed datasets, with a burn-in phase of 5000 and the number of iterations between each imputation set to 1000. To check convergence, the potential scale reduction factor, R̂, was assessed for each variable used in the imputation process. Several auxiliary variables returned R̂ values greater than two indicating potential non-convergence of chains. These variables were dropped from the formula and the imputation process was repeated. Examination of trace and autocorrelation plots for the final imputation attempt indicated that convergence could be assumed (Roy, 2020).

Statistical analyses were all conducted in R, using the lme4 (Bates et al., 2015), nlme (Pinheiro et al., 2021), and mitml packages (Grund et al., 2021). Linear mixed effects modelling (LMM) was used to assess the association of sleep hygiene behaviours with sleep. Day number (i.e., 1–14) of the study is often considered nested within participants as a repeated measured factor due to potential practice/habituation effects occasionally observed across time (Murphy et al., 2022), however in the present research models with day fitted as a random factor showed no improvement over those without. Therefore, the random factor structure of all models consisted of only each participant. Furthermore, the predictors were disaggregated into between and within person components so that estimates generated from each model were accurate representations of the repeated measures data structure (Curran & Bauer, 2011; Wang & Maxwell, 2015).

To determine whether the effect of sleep hygiene behaviours on sleep differed significantly from zero, a null model was constructed which included the demographical variables (gender, age, and sleep disorder) and compared to a model including all sleep hygiene behaviours fitted as individual fixed effects. The estimation method used for each model was maximum likelihood. To assist in model convergence, continuous variables were scaled and centred around the grand mean (Bolker et al., 2009).

To assess whether the effect of each sleep behaviour on each sleep outcome varied according to individual factors, new models were fitted identical to their predecessor except for the inclusion of a random slope for the behaviour of interest.

## Results

### Sample descriptives

The mean age of the participants was 22.12 (*SD* = 5.59), and participants were predominately female (70.3%) with one participant identifying as non-binary. The remaining demographic characteristics are summarised in Table 2, and sleep variable descriptives in Table 3.

**Table 2:** Summary of demographic characteristics (N = 74)

	n	(%)
**Ethnicity**		
Asian	20	(27.0)
Caucasian	49	(66.2)
Other	5	(6.8)
**Sleep Disorder**		
None	69	(93.2)
Insomnia	3	(4.2)
Sleep Apnea	1	(1.4)
**Highest Education Level**		
High School	51	(68.9)
Certificate I-IV	12	(16.2)
Advanced Diploma/Diploma	3	(4.1)
Bachelor’s Degree	7	(9.5)
Graduate Certificate/Diploma	1	(1.4)
**Children Under Two**		
Yes	2	(2.7)
No	72	(97.3)

**Table 3:** Key variable descriptives (N = 1036)

Actigraphy Estimated Sleep Variables	Global Mean	Global SD
Bedtime	11:59 pm	111 min
Waketime	8:09 am	116 min
Sleep Duration	393 min	77 min
Sleep Efficiency	80.39%	8.25%
**Sleep Hygiene Behaviours**	**Mean Nights Performed (out of 14)**	**Percentage (%)**
Pondering Unresolved Matters	3.64	26.0
Exposure to Sunlight (during day)	11.91	85.1
Screen Use	12.63	90.2
High Concentration	2.39	17.1
Negative Emotional States	3.11	22.2
Night-time Worry (about sleep)	1.69	12.1
Unpleasant Conversation	0.67	4.8
Music or TV	2.23	15.9
Checking the Time (during sleep period)	2.46	17.6
Daytime Worry (about sleep)	0.90	6.4
Vigorous Exercise	1.69	12.1
Napping	1.09	7.8
Moderate Exercise	3.89	27.7
Hungry	0.78	5.6
Thirsty	1.48	10.6
Caffeine	1.72	12.3
Other Stimulating Substances	1.18	8.4
Alcohol	2.18	15.6
Too Much Water	0.80	5.7
Too Much Food	0.80	5.7
Noisy or Quiet Environment	1.11	7.9
Bright or Dark Environment	0.99	7.1
Poor Ventilation	0.17	1.2
Uncomfortable Sleep Environment	0.43	3.1
Being Awoken by Partner	1.30	9.3
Social Media Use	10.04	71.7
Sleep Medications	0.76	5.4
Deliberately Using Alcohol to Help Sleep	0.24	1.7
Being Awoken by Pets	1.4	10.3

### Sleep duration

The null model including the demographic variables was constructed, and results indicated that only gender was significantly related to sleep duration (*B* = 24.22, *SE* = 10.85, *t* = 2.23, *p* = .026). Pooled likelihood ratio tests indicated that the model containing gender and all sleep hygiene behaviours provided a significantly better fit over the null model *(F* = 48.357, *df*_1_ = 262, *df*_2_ = 123,700,000, *p* < .001).

The full model (all sleep hygiene behaviours and gender), shown in Table 4, indicated that sleep hygiene behaviours accounted for the gender differences in sleep duration, as gender became non-significant. The estimated sleep duration at the reference level (i.e., when all sleep hygiene behaviours were not performed) was 374 min (6 h 14 min). Random effect estimates indicated total sleep duration for participants varied around the mean sleep duration by 21 min.

**Table 4:** Full linear mixed model of sleep duration being predicted by all sleep hygiene behaviours and gender

Random Effects				
	Group	Variance	*SD*	ICC
	Participant (intercept)	427.05	20.67	0.358
**Fixed Effects (Between)**				
	**B**	***SE***	***t***	***p***
(Intercept)	374.45	27.62	13.56	< 0.001
Gender	2.08	8.11	0.26	0.797
Bedtime*	-41.85^†^	8.12	-5.16	< 0.001
Waketime*	52.81^†^	7.42	7.12	< 0.001
Pondering Unresolved Matters	-19.54	20.60	-0.95	0.343
Exposure to Sunlight (during day)	30.82	18.16	1.70	0.090
Screen Use	20.73	31.21	0.66	0.507
High Concentration	20.50	18.20	1.13	0.260
Negative Emotional States	-12.18	26.20	-0.47	0.642
Night-time Worry (about sleep)	45.08	26.99	1.67	0.095
Unpleasant Conversation	3.25	50.88	0.06	0.949
Music or TV	-21.16	13.34	-1.59	0.113
Checking the Time (during sleep period)	-14.00	18.78	-0.75	0.456
Daytime Worry (about sleep)	-22.49	24.58	-0.92	0.360
Vigorous Exercise	2.04	18.92	0.11	0.910
Napping*	-103.01	31.51	-3.27	0.001
Moderate Exercise	16.97	15.45	1.10	0.270
Hungry	-22.88	30.78	-0.74	0.457
Thirsty	32.31	23.38	1.38	0.167
Caffeine	38.28	19.79	1.93	0.053
Other Stimulating Substances	23.26	25.02	0.93	0.353
Alcohol*	-43.83	20.56	-2.13	0.033
Too Much Water*	66.98	30.04	2.23	0.026
Too Much Food	-41.83	36.79	-1.14	0.256
Noisy or Quiet Environment	-30.43	46.19	-0.66	0.510
Bright or Dark Environment	-3.64	43.86	-0.08	0.934
Humid or Dry Environment	-30.61	100.80	-0.30	0.761
Poor Ventilation	-125.13	100.55	-1.24	0.213
Uncomfortable Bed Environment	-11.45	37.71	-0.30	0.761
Being Awoken by Partner	19.36	21.01	0.92	0.213
Social Media Use*	-32.71	15.17	-2.16	0.031
Sleep Medications	18.93	16.28	1.16	0.245
Deliberately Using Alcohol to Help Sleep	29.20	35.38	0.83	0.409
Being Awoken by Pets	18.87	18.11	1.04	0.297
**Fixed Effects (Within)**				
	**B**	***SE***	***t***	***p***
Bedtime*	-54.97^†^	1.05	-52.58	< 0.001
Waketime*	54.23^†^	1.03	52.75	< 0.001
Pondering Unresolved Matters	-5.25	2.98	-1.76	0.078
Exposure to Sunlight* (during day)	7.22	3.27	2.21	0.027
Screen Use	2.84	4.28	0.66	0.507
High Concentration	3.92	3.12	1.25	0.210
Negative Emotional States	-1.11	3.06	-0.36	0.717
Night-time Worry* (about sleep)	-7.27	3.67	-1.98	0.048
Unpleasant Conversation*	-13.71	5.50	-2.49	0.013
Music or TV	-3.55	4.04	-0.88	0.380
Checking the Time (during sleep period)	-0.54	3.07	-0.18	0.859
Daytime Worry (about sleep)	4.91	5.76	0.85	0.394
Vigorous Exercise	1.31	3.64	0.36	0.718
Napping	4.67	4.07	1.15	0.251
Moderate Exercise	0.42	2.47	0.17	0.865
Hungry	-1.22	5.14	-0.24	0.813
Thirsty	0.86	4.17	0.21	0.836
Caffeine	-2.49	3.73	-0.67	0.505
Other Stimulating Substances	-7.54	6.82	-1.11	0.269
Alcohol	-2.74	3.62	-0.76	0.450
Too Much Water	1.14	5.04	0.23	0.821
Too Much Food	-3.50	4.59	-0.76	0.446
Noisy or Quiet Environment	-0.76	5.97	-0.13	0.898
Bright or Dark Environment	2.40	7.28	0.33	0.742
Humid or Dry Environment	-11.43	10.63	-1.08	0.282
Poor Ventilation*	-22.99	10.20	-2.25	0.024
Uncomfortable Bed Environment	-3.22	7.05	-0.46	0.648
Being Awoken by Partner	-5.96	4.18	-1.42	0.154
Social Media Use	-1.25	3.58	-0.35	0.726
Sleep Medications	-14.52	21.37	-0.68	0.497
Deliberately Using Alcohol to Help Sleep*	55.00	14.95	3.68	<. 001
Being Awoken by Pets	-7.18	4.09	-1.76	0.079

*Note*. Except for bedtime and waketime, between-person effects represent the association of the frequency of performance of sleep hygiene behaviours with mean sleep duration over the course of the 14-day period. Within-person effects represent the specific daily association of performing a behaviour with sleep duration compared to not performing the behaviour. ICC = Intraclass coefficient

^†^As continuous variables, bedtime and waketime were standardised before being entered into the model and therefore standardised coefficients are shown instead

* Denotes significance at the *p* < .05 level

Further examination of the full model indicated that the between-persons effects for bedtime, napping during the day, alcohol consumption, and social media use were all significantly negatively related to sleep duration, whereas waketime and too much water before bed was positively related to sleep duration (Table 4). Within-person effects showed that bedtime, worrying about sleep at night, having an unpleasant conversation before bed, and poor ventilation were significantly negatively related to sleep duration, whereas waketime, exposure to sunlight, and deliberate use of alcohol to assist with sleeping were significantly positively related to sleep duration.

Across all participants, this equated to 22.62 min less sleep for every hour later their mean bedtime was, 7.4 min less sleep for every extra occurrence of a daytime nap, 3.1 min less sleep for every extra occasion of drinking alcohol, and 2.3 min less sleep for every extra use of social media before bed across the 14-day period, as well as an extra 27.3 min more sleep for every hour later their mean waketime was, and 4.8 min more sleep for every extra night that too much water was drunk before bed across the 14-day period.

Within participants, this equated to 29.7 min less sleep for every hour later that their bedtime was with respect to their mean. When compared to nights that they did not complete the behaviour, this equated to 7.3 min less sleep when participants worried about their sleep, 13.7 min less sleep when they had an unpleasant conversation before bed, and 23.0 min less sleep when they slept in poorly ventilated environments. When compared to occasions that they did not, this also equated to 7.2 min more sleep on nights where participants obtained outdoor sunlight exposure during the preceding day, 55.0 min more sleep when they deliberately used alcohol to assist with sleep, and 28.1 min more sleep for every hour later that their waketime was relative to their mean.

Further analyses were conducted to test whether the individual variation between participants (i.e., behaviours that affected some but not others) could explain some of the non-significant findings. Individual models were fitted with random slopes for each behaviour and compared to the model without random slopes to assess improvement. After Bonferroni corrections, pooled likelihood-ratio tests revealed that models with random slopes for going to bed at different times each night *(F* = 8.60, *df*_1_ = 2, *df*_2_ = 527,400, *p* < .001), waking up at different times each morning *(F* = 23.25, *df*_1_ = 2, *df*_2_ = 2679.595, *p* < .001), checking the time during the night *(F* = 6.91, *df*_1_ = 2, *df*_2_ = 11432.15, *p* = .001), and going to bed hungry *(F* = 34.62, *df*_1_ = 2, *df*_2_ = 6374.17, *p* < .001) showed significant improvements over the model without any slopes fitted.

### Sleep efficiency

Given that only 10 behaviours emerged as significant predictors of sleep duration at either the between or within effect level and the majority did not, we considered the possibility that sleep behaviours might be impacting other sleep outcomes instead of sleep duration. For example, it was considered possible that exercising too close to bed may not impact the length of time asleep, but may impact the quality of sleep experienced (e.g., sleep efficiency) as has been demonstrated in certain situations (e.g., among healthy young males; Flausino et al., 2012). Therefore, to assess whether sleep hygiene behaviours were impacting sleep through means other than sleep duration, the same analysis was repeated for sleep efficiency. Although we could have selected any of the other sleep outcomes collected, associations between sleep efficiency and adverse health outcomes (Eiman et al., 2019; Wallace et al., 2021; Yan et al., 2021) appear to be more established than other sleep outcomes making it the next best candidate to assess the impact of sleep hygiene behaviours on sleep.

The null model which included the demographic variables was constructed, and results indicated that none of the variables were significantly related to sleep efficiency. Thus, demographic variables were dropped from subsequent testing of sleep efficiency.

A full model containing all sleep hygiene behaviours indicated that the average sleep efficiency at the reference level (i.e., when all sleep hygiene behaviours were not performed) was 76.75%. Random effect estimates indicated sleep efficiency for participants varied around the mean sleep efficiency by 4.12%. As with sleep duration, only the full model is reported.

Further examination of the full model (Table 5) indicated that between-persons effects for waketime, napping during the day, and alcohol use were all significantly negatively related to sleep efficiency, whereas bedtime, caffeine use, and drinking too much water before bed were all significantly positively related to sleep efficiency (Table 5). Within-persons effects showed that getting exposure to sunlight or outdoor light during the day, and deliberately using alcohol to help fall asleep were both significantly positively associated with sleep efficiency, whereas having an unpleasant conversation before bed was negatively related to sleep efficiency.

**Table 5:** Full linear mixed model of standardised sleep efficiency being predicted by all sleep hygiene behaviours and gender

Random Effects				
	Group	Variance	*SD*	ICC
	Participant (intercept)	17.04	4.13	.360
**Fixed Effects (Between)**				
	**B**	***SE***	***t***	***p***
(Intercept)	76.89	5.41	14.22	< .001
Bedtime*	6.20^†^	1.59	3.89	< .001
Waketime*	-5.13^†^	1.48	-3.47	.001
Pondering Unresolved Matters	-5.16	3.75	-1.38	.169
Exposure to Sunlight (during day)	6.04	3.59	1.68	.092
Screen Use	3.65	6.19	0.59	.555
High Concentration	3.91	3.44	1.14	.256
Negative Emotional States	-1.71	4.90	-0.35	.724
Night-time Worry (about sleep)	8.98	5.04	1.78	.075
Unpleasant Conversation	1.26	9.85	0.13	.898
Music or TV	-4.00	2.50	-1.60	.110
Checking the Time (during sleep period)	-2.46	3.65	-0.67	.501
Daytime Worry (about sleep)	-3.82	4.82	-0.79	.428
Vigorous Exercise	-0.49	3.49	-0.14	.888
Napping*	-22.23	6.28	-3.54	< .001
Moderate Exercise	4.60	3.02	1.51	.131
Hungry	-4.49	6.12	-0.73	.463
Thirsty	6.33	4.66	1.36	.174
Caffeine*	7.93	3.59	2.21	.027
Other Stimulating Substances	5.80	4.75	1.22	.223
Alcohol*	-8.89	3.99	-2.23	.026
Too Much Water*	13.15	5.93	2.22	.026
Too Much Food	-8.67	7.06	-1.23	.219
Noisy or Quiet Environment	-7.17	9.09	-0.79	.430
Bright or Dark Environment	0.35	8.72	0.04	.968
Humid or Dry Environment	-5.77	19.87	-0.29	.772
Poor Ventilation	-24.38	19.65	-1.24	.215
Uncomfortable Bed Environment	-2.48	7.23	-0.34	.732
Being Awoken by Partner	3.41	4.07	0.84	.401
Social Media Use	-5.77	3.01	-1.92	.055
Sleep Medications	3.50	3.24	1.08	.281
Deliberately Using Alcohol to Help Sleep	4.99	7.04	0.71	.479
Being Awoken by Pets	4.04	3.57	1.13	.257
**Fixed Effects (Within)**				
	**B**	***SE***	***t***	***p***
Bedtime	0.37^†^	0.21	1.76	.079
Waketime	-0.07^†^	0.20	-0.35	.725
Pondering Unresolved Matters	-0.87	0.59	-1.46	.144
Exposure to Sunlight (during day)	1.27	0.65	1.96	.050
Screen Use	0.85	0.85	1.01	.314
High Concentration	0.86	0.62	1.38	.168
Negative Emotional States	-0.18	0.61	-0.29	.769
Night-time Worry (about sleep)	-1.18	0.73	-1.61	.108
Unpleasant Conversation*	-2.89	1.09	-2.65	.008
Music or TV	-0.97	0.80	-1.21	.228
Checking the Time (during sleep period)	-0.12	0.61	-0.20	.840
Daytime Worry (about sleep)	1.04	1.15	0.91	.365
Vigorous Exercise	0.31	0.73	0.42	.674
Napping	0.48	0.80	0.59	.553
Moderate Exercise	-0.01	0.49	-0.02	.985
Hungry	0.11	1.02	0.11	.914
Thirsty	0.11	0.83	0.13	.899
Caffeine	-0.49	0.74	-0.66	.510
Other Stimulating Substances	-1.53	1.36	-1.12	.262
Alcohol	-0.46	0.72	-0.63	.526
Too Much Water	0.26	1.00	0.26	.792
Too Much Food	-0.77	0.91	-0.84	.400
Noisy or Quiet Environment	-0.29	1.19	-0.25	.804
Bright or Dark Environment	0.36	1.45	0.25	.806
Humid or Dry Environment	-2.87	2.11	-1.36	.174
Poor Ventilation	-3.54	2.03	-1.75	.081
Uncomfortable Bed Environment	-0.77	1.40	-0.55	.582
Being Awoken by Partner	-1.13	0.83	-1.35	.176
Social Media Use	-0.36	0.71	-0.51	.607
Sleep Medications	-3.59	4.25	-0.85	.398
Deliberately Using Alcohol to Help Sleep*	9.24	2.97	3.11	.002
Being Awoken by Pets	-1.47	0.81	-1.82	.069

*Note*. Except for bedtime and waketime, between-person effects represent the association of the frequency of performance of sleep hygiene behaviours with mean sleep efficiency over the course of the 14-day period. Within-person effects represent the specific daily association of performing a behaviour with sleep efficiency compared to not performing the behaviour. ICC = Intraclass coefficient

^†^As continuous variables, bedtime and waketime were standardised before being entered into the model and therefore standardised coefficients are shown instead

* Denotes significance at the *p* < .05 level

Between participants, this equated to 2.63% lower sleep efficiency for every hour later their mean waketime was, 1.58% lower sleep efficiency for every extra occurrence of a daytime nap, and 0.61% lower sleep efficiency for every extra day that alcohol was consumed over the 14-day period, as well as a greater 3.32% sleep efficiency for every hour later their mean bedtime was, 0.58% greater sleep efficiency for every extra day caffeine was consumed, and 0.93% greater sleep efficiency for every extra occasion where too much water was consumed before bed over the 14-day period.

Within participants, this equated to 1.27% greater sleep efficiency when sunlight exposure was obtained during the preceding day, and 9.24% greater sleep efficiency when alcohol was used deliberately to assist with sleep when compared to nights in which sunlight was not obtained during the day, and nights in which alcohol was not deliberately used, respectively. Furthermore, when compared to nights in which they did not, nights that an unpleasant conversation was had before bed equated to 2.89% lower sleep efficiency.

Further analyses were conducted to test whether the individual variation between participants (i.e., behaviours that affected some but not others) could explain some of the non-significant findings. Individual models were fitted with random slopes for each behaviour and compared to the model without random slopes to assess improvement. After Bonferroni corrections, pooled likelihood-ratio tests revealed that models with random slopes for going to bed at a different time than usual (*F* = 8.63, *df*_1_ = 2, *df*_2_ = 251,900, *p* < .001),waking up at a different time than usual (*F* = 16.57, *df*_1_ = 2, *df*_2_ = 13011.50, *p* < .001), checking the time in the middle of the night (*F* = 7.12, *df*_1_ = 2, *df*_2_ = 83602.68, *p* = .001), and going to bed hungry (*F* = 31.14, *df*_1_ = 2, *df*_2_ = 5237.73, *p* < .001) all showed significant improvements over the model without any slopes.

## Discussion

The findings demonstrated that demographic variables had little association with sleep, in either outcome assessed. Neither age nor previous self-reported diagnoses of sleep disorder were related to either sleep duration or sleep efficiency. Given that age related sleep differences may emerge in samples with a large distribution in age range (Klerman & Dijk, 2008), the comparatively younger age sample in the present study may explain the lack of relationship observed. For the few participants indicating they had sleep disorders, the lack of observable difference may arise from the overall sample consisting of university students, a population with well-established sleep difficulties (Peltzer & Pengpid, 2016), evidenced here in the present study by the average sleep duration (6 h 33 min) which is less than general population estimates (Adams et al., 2017). Gender was the only covariate associated with sleep, and was only related to sleep duration, whereby women experienced slightly longer sleep duration. Contrary to much research suggesting that women experience poorer sleep (Mallampalli & Carter, 2014), this may be an artefact of sleep duration specifically, whereby female students tend to sleep longer when their schedules permit them to (Putilov et al., 2021). This is particularly likely in the present sample given the attenuated relationship between gender and sleep duration in the full model which contained waketime as a predictor.

For the most part, behaviours that were significant contributors to sleep duration are supported by similar research. Later bedtimes are consistently associated with negative sleep outcomes (Li et al., 2018; McMahon et al., 2019) which align with our findings, and later waketimes were also positively associated with sleep, suggesting that those who were able to sleep in later were also able to sleep for longer over the course of the night. Related to the timing of sleep, the consistency of the sleep schedule on a night-by-night basis is a well-established sleep hygiene behaviour (Mastin et al., 2006). When sleep schedules are irregular, such as those experienced by shift workers or university students, poorer sleep quality is often observed (Kang & Chen, 2009; Nena et al., 2018). This was also reflected in our findings which show that within-person effects for both waketime and bedtime predict sleep duration, such that daily fluctuations away from regular sleep schedules were associated with different total nightly sleep durations. Specifically, later than usual bedtime was associated with less overall sleep and later than usual waketime was associated with more overall sleep.

The between-persons effect of napping during the day, alcohol consumption and social media use were also in line with previous research which suggests that more frequent nappers, higher frequency of alcohol consumption, and more frequent social media use are each associated with poorer sleep (Levenson et al., 2016; Mograss et al., 2022; Singleton & Wolfson, 2009). Interestingly, more frequent reporting of having too much water before bed was associated with longer sleep duration, a finding that initially appears to contradict previous research (Grandner et al., 2014). However, given this was a between-persons effect (i.e., reflective of overall occurrences over the course of 14 days as opposed to acute instances of greater water intake) and that Grandner et al. (2014) assessed non-restorative sleep (operationalised as subjective indicators of feeling unrested during the day), it is possible drinking water more consistently is indeed associated with greater overall sleep duration when assessed by actigraphy (Grandner et al., 2010), and that subjective notions of drinking ‘too much water’ might actually reflect individuals who are well hydrated and thus experience flow on health benefits reflected by longer sleep.

Within-persons effects also highlighted associations that were consistent with general sleep hygiene recommendations. This included getting exposure to sunlight or outdoor light during the day (Estevan et al., 2022), having an unpleasant conversation before bed (Yang et al., 2010), and having a poorly ventilated sleep environment (Fritz et al., 2022). However, alcohol when used with the specific purpose to aid sleep was strongly associated with greater sleep duration in the present sample. This is an important finding as it is contrasted with the between-persons effect of general alcohol use. A common cited cause for relapse with alcohol-dependant users are subjective sleep complaints (Landolt & Gillin, 2001; Reid-Varley et al., 2020), and alcohol has been demonstrated to differentially effect sleep such that sleep can improve in the first half of a sleep episode, but deteriorates in later stages (Arnedt et al., 2011; Geoghegan et al., 2012). It is therefore possible that heavier and more dependent users may have been responsible for this phenomenon in our sample whereby greater alcohol consumption overall was related to worse sleep outcomes (between-persons effect), but for acute instances when alcohol was used to intentionally aid in sleep (within-persons effect), longer actigraphy-determined sleep periods were obtained.

Compared to the few behaviours that were significantly associated with sleep duration, there were many more that were not. For some behaviours, a lack of variability is the most likely explanation for null findings, such that infrequent engagement reduced statistical power to detect an effect. For example, overly humid or dry, noisy, or uncomfortable sleep environments occurred less than 10% of the nights across all participants in the present sample. As such, sleep hygiene factors like these may need to be considered in broader samples that capture a larger community cross-section which may not be otherwise represented in a university sample (Social Research Centre, 2022). However, other behaviours like worrying about sleep during the day and items relating to similar ruminative processes which are included in the most prevalent sleep hygiene scales (Lacks & Rotert, 1986; Mastin et al., 2006; Yang et al., 2010), were not associated with sleep duration in the present sample at either the between-persons or within-persons level. Such findings need further exploration.

Random slopes analysis indicated that going to bed hungry had different slope estimates for different individuals, as did going to bed and waking up at a different time to usual and checking the time in the middle of the night. Genetic differences in olfactory receptors have been associated with hunger susceptibility (Choquette et al., 2012), and although the effect of hunger on sleep is less documented, the underlying genetic differences in susceptibility to hunger may contribute to variation in the impacts of hunger on sleep, and would begin to explain the mixed findings of the effect of hunger on sleep in previous work. That the slope of an irregular bedtime varied by participant, suggests that some participants were able to compensate for a change in bedtime better than others by sleeping in or waking up earlier to reach a total sleep period that was more in line with their usual sleep duration, while others were not and consequently experienced larger changes in their usual sleep duration.

The between-person effects for the sleep efficiency model revealed similar findings to that of the sleep duration model, whereby bedtime and waketime were the strongest predictors of sleep efficiency, but frequency of daytime napping, caffeine use, alcohol use, and water intake before bed over the 14-day period were all related to sleep efficiency too. The relationships between the frequency of daytime napping and alcohol use with sleep efficiency are less established than with sleep duration, so the present findings provide further useful insight into their potential impact on sleep, demonstrating that they are negatively associated with sleep outcomes beyond just sleep duration. As with sleep duration, frequency of too much water before bed was also positively associated with sleep efficiency. Counterintuitively however, frequency of caffeine use over the 14 days was weakly and positively related to sleep efficiency. While most evidence tends to align with caffeine negatively impacting sleep (Clark & Landolt, 2017), higher caffeine use in the between-persons effect represents a greater proportion of days in which caffeine was consumed over the 14-day period and therefore research on habitual caffeine users may be more useful in explaining this finding. Indeed, habitual users seem to be less effected by the deleterious effects of caffeine on sleep (LaJambe et al., 2005), showing no improvements in sleep efficiency when they abstain from use (Irish et al., 2021), and receive an overall ‘net benefit’ of caffeine due to overcoming withdrawal symptoms (O’Callaghan et al., 2018). However, this net benefit is used mostly to describe the psychoactive effect of caffeine (e.g., psychomotor vigilance performance) and more systematic work is needed to see if this extends to sleep efficiency as well. Finally, bedtime and waketime were positively and negatively related to sleep efficiency respectively. Both of these associations are in the opposite direction to their associations with sleep duration which demonstrates that although a later bedtime meant less time to sleep, it also meant that there was less time in bed spent awake. Likewise, earlier waketimes meant more efficient, albeit shorter, sleep. More research is needed to clarify whether this was an artefact of conscious changes in bed-related behaviours (i.e., less time in bed doing non-sleep related activities), or whether these behaviours contribute to a more fragmented sleep period (more frequent and longer lasting nocturnal awakenings) resulting in lower sleep efficiency. (Almojali et al., 2017; Mograss et al., 2022; Singleton & Wolfson, 2009)

Within-persons effects for sleep efficiency identified only two behaviours that were significant predictors; having an unpleasant conversation before bed and using alcohol with sleep-assisting intentions. Both of these behaviours may be explained in the same way as they were for the sleep duration findings since alcohol use in this manner was found to be positively related with sleep efficiency, and having an unpleasant conversation before bed was negatively related. Taken together with the sleep duration findings, it seems likely that they both contribute to a change in sleep efficiency, which when total time in bed is held constant, translates into a change in sleep duration.

The lack of emerging significant associations for the remaining variables in sleep efficiency are of potential concern, given there were still many sleep hygiene behaviours that were not associated with sleep at all. Two of these non-significant findings could be attributed to significant variation that was observed between participants. Random slopes models for sleep efficiency highlighted that the same behaviours that varied for sleep duration also varied for sleep efficiency (variable bedtime, variable waketime, checking the time in the middle of the night, and going to bed hungry) underscoring different associations for these behaviours with sleep for different individuals. As described above, variation in the slope of going to bed hungry may be attributed to individual differences in susceptibility to hunger. That the slope of checking the time in the middle of a night varied by participant may also be explained empirically.

Poorer sleepers and those with clinical insomnia tend to display attentional biases with sleep-related stimuli such as clocks (Woods et al., 2009) to determine how long they have left to sleep (Harvey & Tang, 2012) which in turn can elicit arousal (Tang et al., 2007) and maintain wakefulness. Thus, checking the time might only impact sleep for the subset of participants who are prone to engaging in such unhelpful clock monitoring and therefore explain why at the overall level it was not found to be significantly related to sleep (only some participants may be affected by it). Alternatively, personal perceptions about how a specific behaviour may affect one’s sleep (McAlpine et al., in press), attitudes towards sleep (Peach et al., 2018), or personality factors (Duggan et al., 2014) are all possible contributors to the individual variation in effects observed here, and although outside the scope of the present research, represent suitable avenues for future research to elucidate these effects.

It should also be noted that although significant individual fixed effects were not observed for every behaviour, there were marked similarities across several behaviours (e.g., worrying about sleep during the night, worrying about sleep during the day). As such, similar behaviours may share variance in the outcome variable, limiting the likelihood of them all emerging as significant independent predictors. However, summing all within-person sleep hygiene estimates suggest that performance of all behaviours in their hypothesised sub-optimal direction results in considerable decreases in estimates for both sleep duration and sleep efficiency on a given night, highlighting the potential cumulative effects of performing all behaviours the same direction (abstinence vs. performance).

The present research raises several important questions regarding the effect of sleep hygiene behaviours on sleep and contains several strengths. Firstly, the use of actigraphy cross-checked with subjective measures for accuracy, increased the validity of the data, and allowed for a greater degree of confidence in the results. Secondly the use of linear mixed modelling analysis coupled with the repeated measures design, allowed us to control for each association by participant, as well as separate between and within components from the data to provide estimates for each that are instantly transferrable into real-world contexts. For example, within-estimates showed that a poorly ventilated environment on any given night was associated with an average of 23 min less sleep. Similarly, adhering to a consistent sleep schedule, acute sunlight or outdoor light exposure during the day, and avoiding or better managing unpleasant conversations before bed may assist in better sleep outcomes. Between-person effect estimates have also established more concrete support for the overall association of specific behaviours with sleep. Namely, that the frequency of daytime napping, alcohol use, and social media use should be reduced, water should be more regularly consumed, and consideration of the specific timing of the sleep period should be made if increasing sleep duration is a priority. Finally, the inclusion of many different sleep hygiene behaviours in one model allows for estimates that are independent of other effects (i.e., estimates that also control for the likely scenario where multiple inhibitory behaviours are performed in one day).

One obvious limitation, however, pertains to the use of a university-based sample. As such, extrapolation of the results to the general population must be considered with caution. However, given this population experience poorer sleep than other non-clinical populations (Peltzer & Pengpid, 2016), it was a suitable target to identify potentially influential behaviours contributing to poorer sleep. Further research could therefore examine whether these findings apply to other non-clinical populations outside of university settings too. Another potential limitation pertains to the exclusion of items that participants said they never did at baseline, resulting in them never being asked about those items over the observational period. While this was done to reduce participant burden it is possible that participants could have engaged in those behaviours anyway. However, allowing for open-ended additional comments in each daily questionnaire for occasions such as these allowed us to correct for circumstances where this could have occurred, minimising this risk.

## Conclusions

Overall, the findings shed some light on the way in which sleep hygiene behaviours should be considered as impacting sleep moving forward. First and foremost is that the mechanism by which any given sleep hygiene behaviour affects sleep should be considered when making recommendations. For instance, some behaviours are associated with sleep duration, but not efficiency (e.g., social media use), and vice versa (e.g., caffeine use). Thus, the recommendations for the general population wishing to improve their sleep need to be tailored to assess which domains of sleep they are having difficulties with. Secondly, the effect of individual differences should not be discounted when making recommendations. The finding that the association of several behaviours on sleep varied considerably should be accounted for within recommendations. The sources of these individual variations are yet to be conclusively established and further research is needed to explore some of these internal factors. However, recommendations should still look to acknowledge and educate those that wish to adopt them that some behaviours may have more of an influence on some people than others. Therefore, an iterative process may be useful for individuals to determine what works best for them.

Finally, the present findings highlight that many commonly used sleep hygiene recommendations warrant further attention to conclusively establish grounds for inclusion. If future experimental research serves to replicate the present findings in a broader population, the numerous behaviours that were not associated with any sleep outcome, (e.g., falling asleep while watching TV/listening to music, engaging in high levels of concentration before bed, and going to bed thirsty) may need to be dropped from recommendations to ensure that guidelines accurately reflect observed data.

## Data Availability

The data underlying this article cannot be shared publicly due to the conditions under which ethics were obtained. Participants were informed that only “The following people will have access to the information we collect in this research: the research team and, in the event of an audit or investigation, staff from the Curtin University Office of Research and Development”. As such we are unable to make data publicly available.
